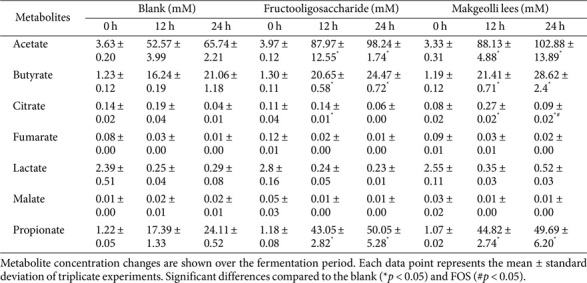# Makgeolli Lees as a Novel Prebiotic Candidate: Effects on Human Gut Microbiota and Metabolites

**DOI:** 10.4014/jmb.2504.04020

**Published:** 2025-06-23

**Authors:** Jun Hoi Kim, Hwa Rin Kim, Hyunbin Seong, Seung Hee Han, Geonhee Kim, Yuri Choi, Nam Soo Han

**Affiliations:** 1 Brain Korea 21 Center for Smart GreenBio Convergence and Sustainable Regional Development, Division of Animal, Horticultural, Food Sciences, Chungbuk National University, Cheongju 28644, Republic of Korea; 2 Gaesinbiotech, Cheongju 28644, Republic of Korea

**Keywords:** Makgeolli lees, prebiotics, in vitro fecal fermentation, gut microbiota, short-chain fatty acids

## Abstract

Makgeolli lees (ML), an underutilized byproduct of traditional Korean rice wine production, contains abundant indigestible carbohydrates and microbial residues. This study evaluates the prebiotic potential of ML using a simulated human digestion and *in vitro* fecal fermentation model. *In vitro* digestion showed that 61.30% of total carbohydrates and 89.92% of crude protein remained undigested. During *in vitro* fecal fermentation, treatment with ML induced compositional changes in the gut microbiota, including increased abundances of *Bifidobacterium bifidum* (Δ 0.37 ± 0.09%), *Prevotella copri* (Δ 0.98 ± 0.24%), and *Bacteroides uniformis* (Δ 0.46 ± 0.06%), along with a decrease in *Bacteroides ovatus* (Δ –2.14 ± 1.51%). In addition, SCFA concentrations were elevated after 12 h of fermentation, with acetate at 84.80 ± 4.88 mM, propionate at 43.75 ± 2.74 mM, and butyrate at 20.22 ± 0.71mM. The alterations in microbial composition and associated metabolite profiles were comparable to those observed following fructooligosaccharides (FOS) supplementation. These findings indicate that ML function as a fermentable substrate that selectively enriches health-associated gut microbiota and enhances the production of beneficial microbial metabolites, thereby supporting their potential application as a sustainable and cost-effective prebiotic candidate in dietary applications.

## Introduction

Makgeolli is a traditional Korean fermented rice beverage that is produced through a two-stage fermentation process using nuruk, a natural starter containing fugal enzymes such as amylase and protease [1, 2]. In the first step, steamed rice is hydrolyzed by *nuruk* enzymes, and in the second step, alcoholic fermentation occurred by yeast [3]. After fermentation, the mixture was diluted to approximately 6% alcohol concentration and bottled for distribution. Makgeolli is a milky, off-white, and lightly sparkling rice wine with a characteristic viscosity and a flavor profile that combines mild sweetness and creaminess with subtle carbonation. During storage, rice solids gradually settle at the bottom, leading to a phase separation between a clear supernatant and a cloudy sediment. Depending on preference, consumers either shake the bottle and consume the cloudy suspension or drink only the clear supernatant. In this study, the settled, insoluble fraction is defined as makgeolli lees (ML).

ML contain unfermented rice components, organic acids, ethanol, and a variety of nutrients such as vitamins, minerals, and dietary fiber [4]. It is also rich in yeast and lactic acid bacteria derived from the fermentation process. Several studies have reported antioxidant, anti-thrombotic, and hepatoprotective activities associated with ML [5, 6], supporting their potential use as a functional ingredient in food applications. Hydrolyzed lees have been employed as carbon sources for microbial production of polyhydroxyalkanoates [5], and dietary fiber extracted from lees has been applied to reduced-fat meat products to improve texture and nutritional composition [7]. Although the functional properties have been partially characterized, the prebiotic effects of ML remain largely unexplored. The high content of indigestible components suggests potential for fermentation by gut microbiota, which may contribute to modulation of the intestinal environment.

The human gut contains a diverse microbial population that plays an essential role in nutrient metabolism, immune modulation, and protection against pathogens [8, 9]. Among the major microbial metabolites, short-chain fatty acids (SCFAs) such as acetate, propionate, and butyrate are produced through microbial fermentation of dietary substrates and contribute to maintenance of intestinal homeostasis [10]. These metabolites support epithelial barrier integrity and exhibit anti-inflammatory properties. Prebiotics are defined as substrates selectively utilized by host microorganisms to confer health benefits [11]. Representative prebiotics include fructooligosaccharides (FOS), galactooligosaccharides (GOS), and inulin, which have been widely studied for their ability to stimulate the growth of beneficial bacteria such as *Bifidobacterium* and Lactobacillus [12]. However, the industrial production of purified prebiotics often requires high production cost and complex purification steps, which may limit their practical application [13]. To address this limitation, food processing byproducts have been proposed as alternative prebiotic sources due to their low cost and sustainability [14, 15]. Recent studies have highlighted that various food processing byproducts rich in indigestible carbohydrates, including brewers’ spent grain, cheese whey, and yogurt acid whey, can serve as sustainable prebiotic substrates by promoting beneficial gut microbial shifts and enhancing SCFA production during fermentation [16-18]. These byproducts contain resistant polysaccharides, dietary fibers such as arabinoxylans and β-glucans, and fermentation-derived microbial residues that contribute to their prebiotic effects. In this context, ML, which are rich in unfermented rice polysaccharides, β-glucans, and microbial cell wall components, also possess compositional characteristics favorable for prebiotic application. Therefore, the valorization of ML as a fermentable and sustainable prebiotic substrate holds promise for gut health improvement and circular food system development.

*In vivo* models are commonly used to evaluate dietary effects on the gut microbiota, although they have limitations such as ethical constraints, high cost, and biological variability [19, 20]. In contrast, *in vitro* gut models offer a reproducible and controllable system that mimics human gastrointestinal conditions [21]. These systems allow precise control of pH, temperature, anaerobic conditions, and retention time. Microbial composition and metabolic activity, including SCFA production, can be directly measured under defined conditions [22]. Static batch fermentation models are widely used for screening prebiotic candidates, as they enable assessment of substrate fermentability and microbial shifts within a short timeframe [23]. *In vitro* fermentation provides an efficient platform for evaluating the gut microbiota-modulating effects of dietary substrates, especially during the early stages of functional food development.

This study aimed to evaluate the prebiotic effects of ML using a simulated human gut model. *In vitro* gastrointestinal digestion was performed to simulate gastric and intestinal enzymatic conditions. Low molecular weight digestion products were removed by dialysis, and the remaining fraction was freeze-dried. The digested ML were then fermented *in vitro* using human fecal microbiota under anaerobic conditions. Changes in gut microbiota composition were analyzed through 16S rRNA metagenome sequencing. Additionally, SCFAs and other organic acids produced during fermentation were quantified. A blank control without any added carbon source was included to provide a baseline for microbial growth, thereby allowing accurate evaluation of substrate-specific fermentation effects.

## Materials and Methods

### Makgeolli Lees (ML) and Materials

**Fig. 1:**
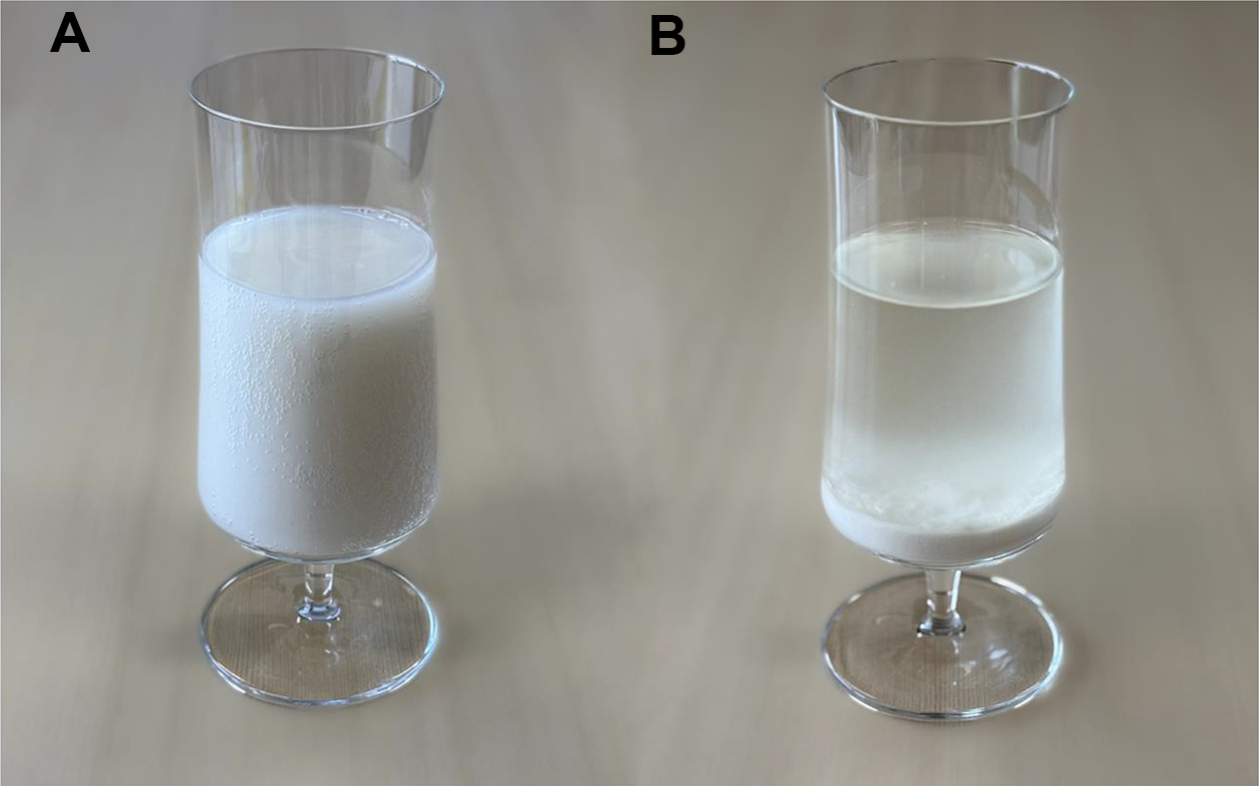
Representative images of makgeolli exhibiting phase separation. Makgeolli was shaken to obtain an evenly suspended cloudy mixture (**A**), while a transparent upper layer was formed under static conditions (**B**).

To minimize product variability, a commercially available rice makgeolli (Kooksoondang, Republic of Korea) manufactured through a traditional and standardized fermentation process was selected. To prepare ML, the product was settled for 3 h without agitation to allow phase separation (Fig. 1A), and the clear supernatant layer (Fig. 1B) was carefully removed. The remaining sediment was collected and centrifuged at 7,000 ×*g* for 10 min. The pellet was then lyophilized and ground into a powder for further analysis. Pepsin (from porcine gastric mucosa, 3,200–4,500 U/mg protein), pancreatin (from porcine pancreas), bile salts, CaCl_2_·2H_2_O, KH_2_PO_4_, NaCl, NaHCO_3_, KCl, MgCl_2_·6H_2_O, (NH_4_)_2_CO_3_, FeSO_4_·7H_2_O, mucin (type III, porcine stomach), glucose, and starch were purchased from Sigma-Aldrich (USA). Peptone water, yeast extract, tryptone, and hemin were obtained from BD Difco (USA). Casein was from Junsei (Japan). Vitamins and cofactors including thiamine, pantothenate, nicotinamide, biotin, cyanocobalamin, para-aminobenzoic acid, and menadione were from Wako (Japan). Tween 80 was purchased from VWR (Radnor, USA). All chemicals used were of analytical grade.

### *In Vitro* Digestion

*In vitro* digestion was conducted with slight modifications based on a previously reported protocol [24]. Freeze-dried ML powder (20 g) was hydrated in 25 ml of distilled water (w/v). To simulate the gastric phase under static conditions, 45 ml of simulated gastric fluid (SGF), consisting of 0.5 g/l KCl, 0.12 g/l KH_2_PO_4_, 2.1 g/l NaHCO_3_, 2.76 g/l NaCl, 0.02 g/l MgCl_2_·6H_2_O, 0.04 g/l (NH_4_)_2_CO_3_, and 0.02 g/l CaCl_2_·2H_2_O, was combined with 45 ml of hydrated ML in a 1:1 ratio (v/v). The pH was adjusted to 3.0, followed by the addition of pepsin (2,000 U/ml) and CaCl_2_·2H_2_O. The mixture was incubated in a shaking incubator at 37°C and 150 rpm for 2 h. Subsequently, to simulate the intestinal phase, 90 ml of simulated intestinal fluid (SIF), composed of 0.5 g/l KCl, 0.1 g/l KH_2_PO_4_, 7.14 g/l NaHCO_3_, 2.24 g/l NaCl, 0.07 g/l MgCl_2_·6H_2_O, and 0.09 g/l CaCl_2_·2H_2_O, was added to the gastric-phase treated sample in a 1:1 ratio (v/v). The pH was adjusted to 7.0, and pancreatin (200 U/ml), bile salts, and CaCl_2_·2H_2_O were introduced. The mixture was incubated under the same conditions (37°C, 150 rpm) for an additional 2 h. Following the intestinal phase, the digested solution was transferred into a dialysis membrane with a molecular weight cut-off (MWCO) of 500 Da. The membrane was uniformly coated with polyethylene glycol (PEG) powder and left for 2 h, then immersed in 10 mM NaCl solution for 12 h to simulate the intestinal absorption process. The dialyzed sample was subsequently freeze-dried for further use in *in vitro* fecal fermentation.

### *In Vitro* Fecal Fermentation

*In vitro* human fecal fermentation of ML was performed following an established protocol [25]. More specifically, 0.5 g of digested ML was mixed with 50 ml of basal growth medium. Then, 15 ml of a 10% (w/v) human fecal slurry, prepared by mixing and homogenizing freshly voided adult feces in 0.1 M PBS (pH 7.0), was added to the mixture. The final composition of the basal growth medium was N-acetylglucosamine (1 g/l), alginate (1 g/l), starch (1 g/l), pectin (citrus) (1 g/l), guar gum (1 g/l), xylan (oat spelt) (1 g/l), arabinogalactan (larch wood) (1 g/l), inulin (1 g/l), mucin (porcine gastric type III) (2 g/l), peptone water (5 g/l), yeast extract (4.5 g/l), cysteine (0.8 g/l), tryptone (5 g/l), casein (3 g/l), NaCl (4.5 g/l), KH_2_PO_4_ (0.5 g/l), MgSO_4_·7H_2_O (1.25 g/l), CaCl_2_·2H_2_O (0.15 g/l), NaHCO_3_ (1.5 g/l), bile salt (0.4 g/l), FeSO_4_·7H_2_O (0.005 g/l), KCl (4.5 g/l), thiamine (4 μl/l), pantothenate (10 μl/l), nicotinamide (5 μl/l), biotin (2 μl/l), cyanocobalamin (0.5 μl/l), para-aminobenzoic acid (5 μl/l), menadione (1 μl/l), hemin (0.05 g/l), and Tween 80 (1 ml/l). Fecal samples were obtained from 10 healthy adult volunteers (age 25–30) who had not taken antibiotics, prebiotics, or probiotics and had no recent history of gastrointestinal disorders. To minimize inter-individual variability, the samples were pooled to generate a representative inoculum for fermentation. The experimental protocol was approved by the Institutional Review Board of Chungbuk National University (IRB No. 2023024431). During fermentation, the samples were continuously stirred, and the pH and temperature were maintained at 6.8 and 37°C, respectively, in an anaerobic chamber (Coy Laboratory Products Inc., USA). Fermentation samples (10 ml) were collected at 12 and 24 h for microbiome and metabolite analysis.

### Proximate Analysis of ML

The total carbohydrate content of ML was determined using a phenol sulfuric acid method [26]. Samples, both before and after *in vitro* digestion, were diluted to a concentration of 0.05 mg/ml. Soluble starch was used as a positive control. Each sample was mixed with 5% phenol in a 1:1 ratio, followed by the addition of sulfuric acid. The mixture was incubated at 25°C for 10 min, and absorbance was measured at 470 nm. The total carbohydrate content was calculated using a glucose standard curve. The crude protein content of ML was analyzed using a modified Kjeldahl method based on a previously established procedure [27]. ML samples (1 g each), both before and after *in vitro* digestion, were wrapped in filter paper and placed in Kjeldahl reaction tubes. A catalyst mixture (CuSO_4_:K_2_SO_4_, 1:9) and 15 ml of sulfuric acid were added, and the samples were subjected to digestion for 3.5 h. Following digestion, the samples were alkalized to convert ammonium ions into ammonia, which was subsequently distilled and collected. The ammonia concentration was determined by titration with 18% 1N NaOH to calculate the nitrogen content. The crude fat content of ML was quantified through Soxhlet extraction, following a previously reported method with minor modifications [28]. ML samples (5 g each), both before and after *in vitro* digestion, were dried at 95–105°C, cooled in a desiccator, and reweighed. The dried samples were extracted with ether under reflux at 60°C for 2 h. Upon completion, the ether was evaporated, and the samples were further dried at 95–105°C for 1 h before cooling and reweighing to determine the crude fat content.

### Metagenome Analysis

The microbial communities in the *in vitro* fecal fermentation samples were analyzed using tag-encoded 16S rRNA gene-based Illumina MiSeq platform (Illumina, USA). The 16S rRNA gene was amplified using a primer set of 341-F (5'-CCTACGGGNGGCWGCAG-3') and 785-R (5'-GACTACHVGGGTATCTAATCC-3') [29] compatible with the Nextera Index Kit (Illumina). Raw sequences were trimmed using a Cut Adapter and analyzed using Deblur pipeline in QIIME2 [30, 31]. Classification was performed using a pre-trained Naive Bayes classifier and the weighted SILVA 132 as a reference database (https://www.arb-silva.de/documentation/release-132/). The alpha diversity was calculated using QIIME2. The specific metrics calculated included observed OTUs, Shannon index, Evenness, and Chao1 index.

### Metabolite Analysis

Changes in metabolite concentrations in fermented samples were analyzed using ¹H-NMR [32]. In brief, fermented samples were centrifuged at 16,000 ×*g* for 10 min. The supernatant was mixed with an equal volume of deionized water containing 10% D_2_O and 1 mM DSS, yielding a final DSS concentration of 0.5 mM. The pH of the mixture was adjusted to 6.00 ± 0.01 using 2 M HCl or NaOH. An aliquot (700 μl) of the mixture was transferred to 5 mm NMR tubes (Norell, USA), and ¹H-NMR spectra were acquired on a Varian INOVA 500 MHz NMR spectrometer (Varian Inc., USA). Identification and quantification of metabolites were performed using the Processor and Profiler modules of the Chenomx NMR Suite v6.1 (Chenomx Inc., Canada).

### Statistics

Each experiment was conducted in triplicate, and the data are presented as the mean ± standard deviation (SD). Statistical analyses were performed using GraphPad Prism (version 8.0). Independent *t*-tests were used to compare two groups. The comparisons included ML vs. blank, FOS vs. ML, and FOS vs. blank. The *p*-values less than 0.05 and 0.001 were considered statistically significant.

## Results

### Compositional Changes of Makgeolli Lees (ML) after Digestion

Proximate analysis was conducted to determine the compositional changes in ML before and after *in vitro* digestion. Total carbohydrates were analyzed using the phenol-sulfuric acid method, crude protein was quantified *via* the Kjeldahl method, and crude fat was measured using the Soxhlet extraction method (Table 1). Initially, ML contained 14.59 ± 1.74 g of total carbohydrates, 4.82 ± 0.04 g of crude protein, and 0.16 g of crude fat (per 20.00 g dry weight). After *in vitro* digestion, the total carbohydrate, crude protein, and crude fat contents decreased to 6.85± 0.44 g, 2.81 ± 0.03 g, and 0.13 g, respectively, for the digested residue (12.17 g dry weight). A substantial degradation of carbohydrates was observed, with a 38.70 ± 6.52 g reduction, whereas the decreases in crude protein (10.08 ± 0.15 g) and crude fat (0.15%) were comparatively minor. The pronounced loss of carbohydrates is attributed to enzymatic hydrolysis, where digestive enzymes break down polysaccharides into absorbable monosaccharides. In contrast, the relatively moderate decline in protein content suggests partial hydrolysis into peptides and amino acids, which may be further utilized by gut microbiota during subsequent fermentation. Meanwhile, the negligible change in crude fat content indicates that lipids remained largely unaffected under the given digestive conditions. Crude fat analysis was performed in a single replicate due to the large sample mass required for Soxhlet extraction and the limited volume available after *in vitro* digestion and dialysis. Although this limitation should be taken into account, the digestion results suggest that ML retained a notable portion of indigestible carbohydrates and proteins that could serve as fermentation substrates during *in vitro* fecal fermentation.

### Changes in the Gut Microbiota by ML

**Fig. 2:**
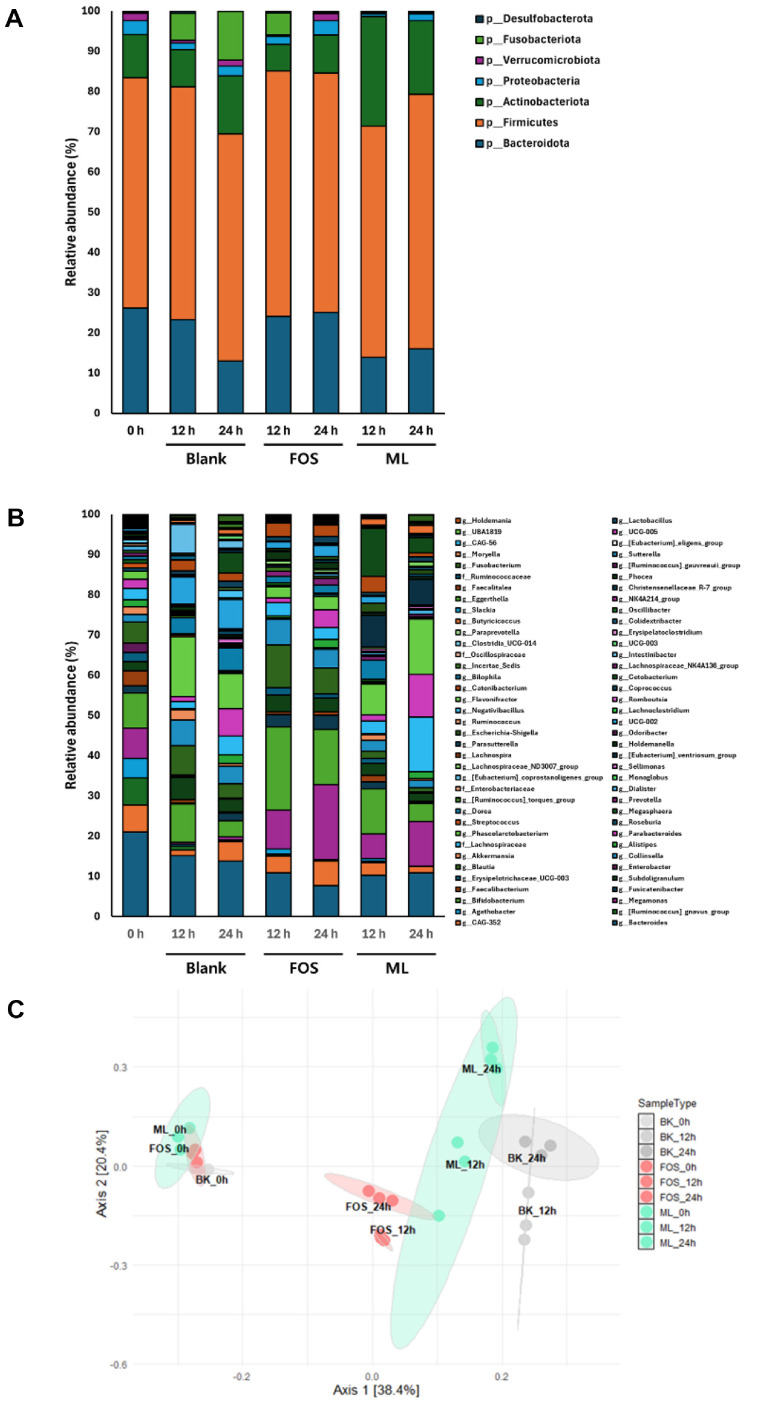
Changes in gut microbiota during fecal fermentation. Changes of microbial abundance were presented at phylum level (**A**) genus level (Among the 108 detected genera, the 41 genera with a relative abundance of 1% or higher were included in the analysis) (**B**) and in its PCoA plot (**C**) during *in vitro* fecal fermentation of fructooligosaccharide (FOS) and makgeolli lees (ML). Samples were collected for metagenomic analysis at 12 and 24 h. A blank group (BK) had no carbon source in the medium.

To investigate the effects of ML on human colonic microbiome composition, an *in vitro* fecal fermentation was conducted following the simulated gastrointestinal digestion. Taxonomic analysis based on 16S rRNA gene amplicon sequencing was performed to assess microbial composition and diversity. As shown in Fig. 2A, at the phylum level, the blank (no-substrate) showed increased Firmicutes (58.65 ± 1.23%) and Proteobacteria (15.45 ± 0.62%), accompanied by decreased Bacteroidetes (13.45 ± 0.53%) and Actinobacteria (2.80 ± 0.14%) after 24 h of fermentation. In contrast, both FOS and ML exhibited increased Actinobacteria (24.35 ± 1.56% and 22.35 ± 0.78%, respectively), with reductions in Firmicutes (46.55 ± 0.98% and 52.45 ± 1.13%) and Bacteroidetes (10.91 ± 0.42% and 15.15 ± 0.65%, respectively). Further taxonomic profiling analysis was conducted to evaluate microbial changes at the genus level during fermentation. As shown in Fig. 2B, *Bifidobacterium* (20.68 ± 0.15%), Megamonas (9.64 ± 0.78%), and *Roseburia* (0.34 ± 0.07%) increased at 12 h in the FOS compared to 0 h (8.70 ± 0.86%, 7.60 ± 0.86%, and 0.83 ± 0.12%, respectively), but decreased at 24 h (13.63 ± 0.9%, 18.70 ± 0.71%, and 0.48 ± 0.18%, respectively). In the ML, *Bifidobacterium* (11.23 ± 0.90%), Megamonas (6.05 ± 0.31%), and *Roseburia* (0.37 ± 0.07%) also increased at 12 h relative to 0 h, but decreased at 24 h (4.46 ± 0.67%, 11.07 ± 0.88%, and 0.05 ± 0.08%, respectively). In the blank, *Bifidobacterium* (9.58 ± 1.20%) showed a slight increase at 12 h compared to 0 h (8.70 ± 0.86%), whereas Megamonas (0.43 ± 0.09%) and *Roseburia* (0.04 ± 0.03%) decreased from their initial values (7.60± 0.86% and 0.83 ± 0.12%, respectively). At 24 h, the abundance of *Bifidobacterium* (4.02 ± 0.18%), Megamonas (0.70 ± 0.06%), and *Roseburia* (0.26 ± 0.09%) in the blank remained lower than their initial levels, indicating minimal recovery. Additionally, principal coordinates analysis (PCoA) was conducted using relative microbial abundances after *in vitro* fermentation. As shown in Fig. 2C, the PCoA plot exhibited distinct clustering of the FOS and ML, which showed similar microbial community patterns at 12 h and 24 h. In contrast, the blank displayed a different distribution with only minor shifts in microbial composition. These results indicate that the FOS and ML induced distinct clustering patterns and more substantial shifts in the gut microbial profile compared to the blank.

### Alpha Diversity Analysis

**Fig. 3:**
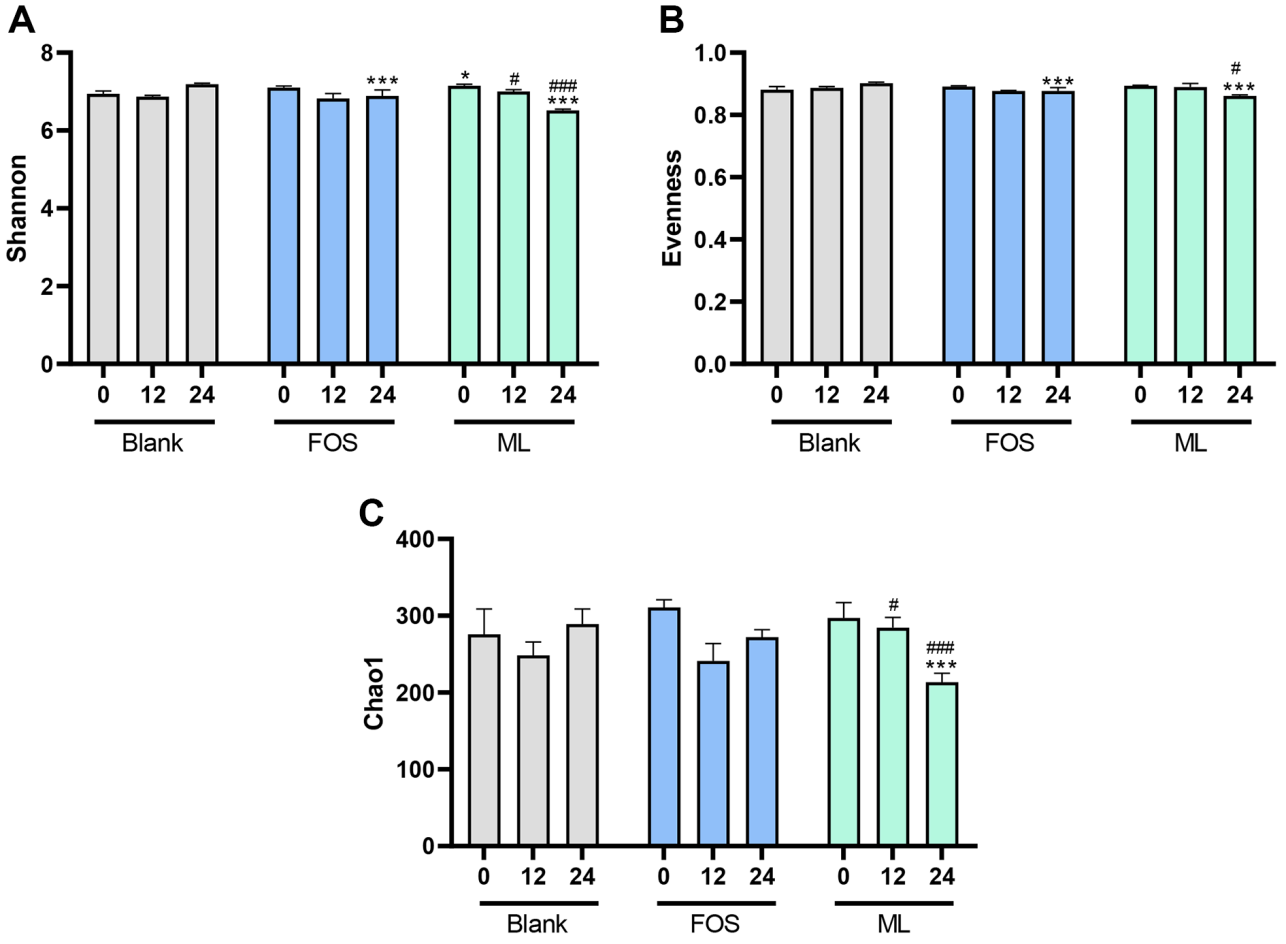
Alpha diversity indices of microbiome data after *in vitro* fecal fermentation of fructooligoasaccharide and makgeolli lees. Alpha diversity was evaluated using (**A**) Shannon index, (**B**) Evenness, and (**C**) Chao1. Fructooligosaccharide (FOS) and makgeolli lees (ML) were subjected to *in vitro* fecal fermentation to assess their impact on microbial diversity. Each data point represents the mean ± standard deviation of triplicate experiments. Significant differences compared to the blank (*p < 0.05 and **p < 0.001), and FOS (#*p* < 0.05 and ###*p* < 0.001).

To evaluate the effects of ML on the α-diversity of microbial communities following *in vitro* fecal fermentation, the Shannon index, Evenness, and Chao1 index were analyzed based on metagenomic abundance. The Shannon index and Evenness reflect microbial distribution by considering the proportional abundance of operational taxonomic units (OTUs), while the Chao1 index estimates species richness based on observed OTUs. As shown in Fig. 3, the Shannon index and Evenness were decreased in the FOS and ML at 12 h and 24 h. For Chao1, ML showed a higher index at 12 h compared to the FOS. However, at 24 h, the index was lower than that of the FOS. The decline in alpha diversity was attributed to the increased relative abundance of dominant taxa such as Bifidobacterium and Megamonas, which were selectively enriched during fermentation.

### Changes in Health-Related Gut Microbiome

To further specify the microbial compositional shifts induced by ML, a species-resolved analysis was performed using delta (Δ) values that reflect changes in relative abundance while accounting for baseline fluctuations. These Δ values were calculated as:

*Δ =(Abundance at 12 h in FOS or ML - Abundance at 0 h in FOS or ML) - (Abundance at 12 h in Blank -Abundance at 0 h in Blank)*

**Fig. 4:**
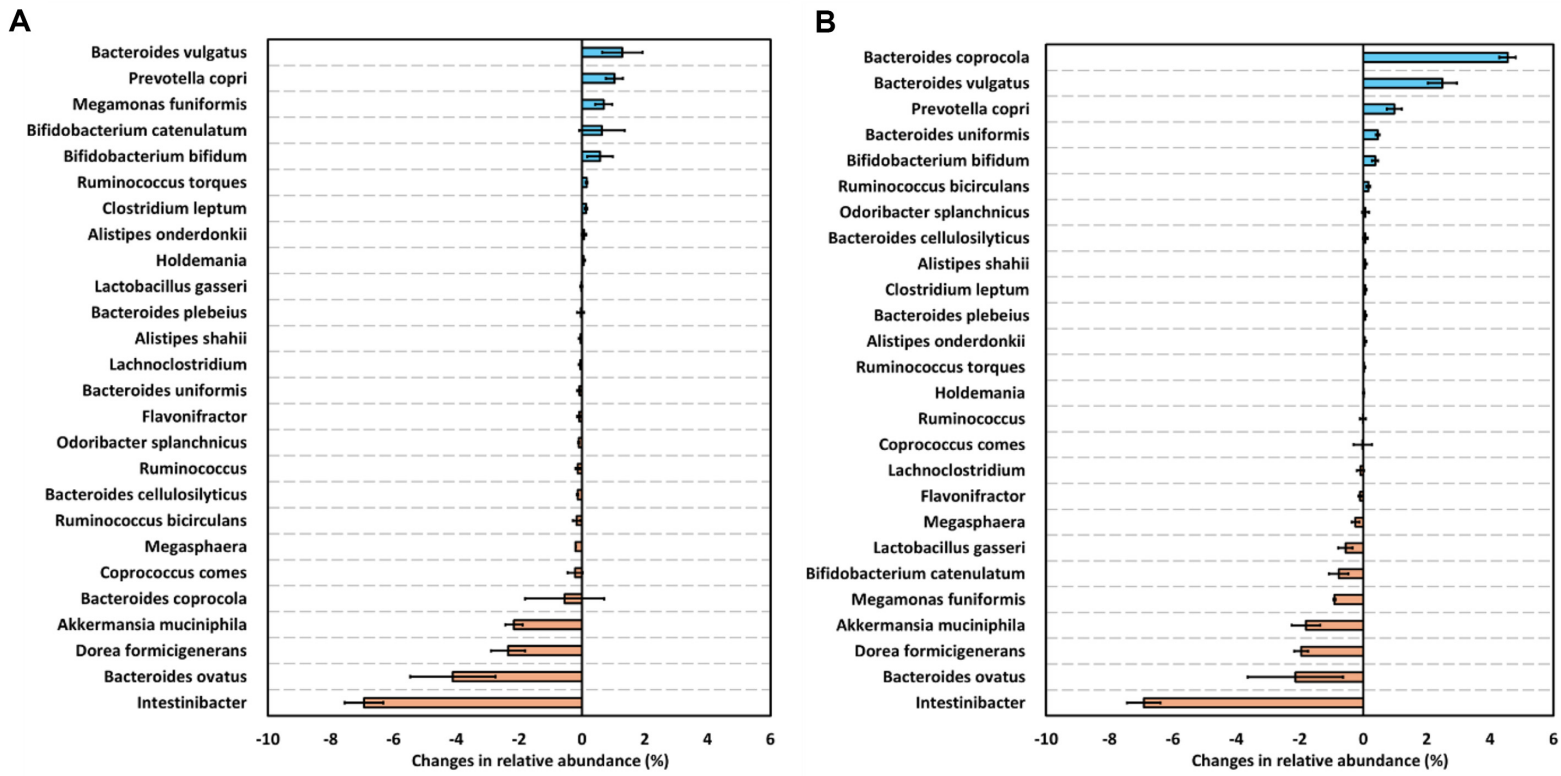
Changes of core health related gut microbial after 12 h *in vitro* fecal fermentation with (A) fructooligoasaccharide and (B) makgeolli lees. Microbial changes in relative abundance are expressed as delta values calculated as (abundance of individual microorganisms in the sample with fructooligosaccharide (FOS) or makgeolli lees (ML) at 12 h − abundance of individual microorganisms in the sample with FOS or ML at 0 h) − (abundance of individual microorganisms in the blank at 12 h − abundance of individual microorganisms in the blank at 0 h). (**A**) represents samples with FOS supplementation, and (**B**) represents samples with ML supplementation during *in vitro* fecal fermentation.

A total of 49 health-positive (H (+)) and 46 health-negative (H (−)) species were defined in a previous study that classified gut microbial taxa based on their health associations [33]. Additionally, 19 probiotic strains formally notified by the Korean Ministry of Food and Drug Safety (MFDS) were included in the consideration. Fig. 4 provides a visual overview of species-level trends, and Table 2 and Table S1 present the detailed quantitative values. In FOS, several beneficial microbes, including *Prevotella copri* (Δ 1.03 ± 0.28%), *Megamonas funiformis* (Δ 0.70 ± 0.27%), and *Bifidobacterium catenulatum* (Δ 0.63 ± 0.73%). *Bifidobacterium bifidum* (Δ 0.57 ± 0.41%) and *Ruminococcus torques* (Δ 0.15 ± 0.02%) also showed positive shifts. In terms of harmful species, *Bacteroides ovatus* exhibited a substantial reduction (Δ –4.11 ± 1.34%), while *Bacteroides vulgatus* showed a modest increase (Δ 1.29± 0.64%). In the ML (Fig. 4B), a comparable enrichment of beneficial species was observed. *Prevotella copri* (Δ 0.98± 0.24%), *Bifidobacterium bifidum* (Δ 0.37 ± 0.09%), and *Bacteroides uniformis* (Δ 0.46 ± 0.06%) were notably elevated. Among harmful species, *Bacteroides vulgatus* showed a mild increase (Δ 2.49 ± 0.46%), while *Bacteroides ovatus* showed a notable decrease (Δ –2.14 ± 1.51%). Interestingly, although generally considered beneficial, *A. muciniphila* and *Dorea formicigenerans* consistently decreased under both treatments, with Δ values of –1.80 ± 0.45% and –1.96 ± 0.21% in the ML, and –2.16 ± 0.28% and –2.35 ± 0.54% in the FOS, respectively. These results suggest that ML selectively enriches health-associated species while limiting the proliferation of potentially harmful taxa. This microbial modulation may contribute to the overall prebiotic potential of ML in fostering a balanced gut environment.

### Changes in Short-Chain Fatty Acid Synthesis during *In Vitro* Fecal Fermentation

To evaluate the effects of ML on SCFA and organic acid production during *in vitro* fecal fermentation, metabolic changes were analyzed using ¹H-NMR over the fermentation period. As presented in Table 3, fermentation of the ML and FOS resulted in significant changes (*p* < 0.05) in the concentrations of acetate, propionate, butyrate, and citric acid compared to the blank. FOS showed larger increases of 84.00 ± 12.55 mM in acetate, 41.87 ± 2.82 mM in propionate, 19.35 ± 0.58 mM in butyrate, and a slight increase of 0.03 ± 0.01 mM in citric acid. Similarly, ML demonstrated significantly higher changes than the blank, with increases of 84.80 ± 4.88 mM in acetate, 43.75 ± 2.74 mM in propionate, 20.22 ± 0.71 mM in butyrate, and 0.19 ± 0.02 mM in citric acid. This metabolic shift may be attributed to microbial changes induced by ML, which likely promoted the growth of SCFA-producing bacteria such as *Bifidobacterium* and Bacteroides (Fig. 2A). In conclusion, the consumption of ML during gut fermentation stimulated the production of beneficial metabolites, such as SCFAs, through the metabolic activity of gut microorganisms, potentially contributing to improved gut health.

## Discussion

The global demand for grain-based alcoholic beverages and their associated fermentation starters has continued to rise in recent years. According to Korean domestic sales data, the makgeolli market grew by 16% over the past five years, from 285 million USD in 2018 to 331 million USD in 2023 [34]. This increased production of traditional rice-based beverages, including makgeolli, yakju, and sake, has drawn attention to the nutritional and functional components generated during fermentation, such as lees. Although makgeolli lees (ML) have been utilized in various applications including animal feed, food additives, and bioresource production [5, 7, 35], their value remains underexplored in the context of human health. In this context, evaluating the prebiotic potential of ML provides a scientific basis for their use as value-added functional ingredients capable of modulating the gut microbiota and promoting intestinal health. To investigate this, we conducted *in vitro* digestion and fecal fermentation experiments. The effects of ML on gut microbiota and SCFA production were assessed using a blank group without a fermentable substrate and FOS as a positive control, thereby validating its potential as a functional dietary ingredient. Accumulating evidence indicates that fermentation byproducts such as sake lees and rice bran exhibit gut microbiota-modulating properties, largely attributed to their content of dietary fibers, β-glucans, and resistant proteins [36-38]. In this study, the ML demonstrated prebiotic activity by increasing the abundance of beneficial microorganisms and decreasing the proportion of potentially harmful bacteria at multiple taxonomic levels (Figs. 2-4, Table 2). At the phylum and genus levels (Fig. 2), supplementation with ML led to an increase in Actinobacteria, particularly *Bifidobacterium*. A similar pattern has been reported with the use of rice bran and sake lees, which support the growth of saccharolytic and anaerobic commensals [36, 39]. At the species level (Fig. 4, Table 2), representative health-associated taxa such as *Prevotella copri*, *Bifidobacterium catenulatum*, and *B. bifidum* were notably enriched following ML fermentation. *A. muciniphila*, a mucin-degrading bacterium involved in intestinal barrier function and host metabolic regulation, exhibited a reduced relative abundance after fermentation. This species utilizes mucin-derived glycans such as N-acetylglucosamine (GlcNAc) as essential growth substrates [40, 41]. The fermentation medium used in this study did not contain mucin or its monomeric components, and the absence of these substrates likely limited the proliferation of *A. muciniphila*. The observed decrease is therefore attributed to substrate unavailability rather than a direct effect of ML. Further investigation using mucin-supplemented media or *in vivo* models is required to evaluate the response of *A. muciniphila* under conditions that better reflect the intestinal environment. Moreover, administration of ML resulted in decreased Shannon index and evenness, indicative of reduced alpha diversity. Alpha diversity, representing the richness and evenness of microbial communities, has been widely regarded as an indicator of gut health. However, recent studies suggest that reduced alpha diversity may not necessarily reflect negative outcomes and can occur due to selective enrichment of beneficial microbes following intake of certain prebiotics or probiotics. For instance, prebiotic supplementation significantly altered specific microbial taxa without globally increasing alpha diversity. Despite this, beneficial immune markers such as reduced CXCL11 levels were observed, suggesting positive health outcomes independent of microbial diversity metrics [42]. Similarly, other studies have shown that increased abundance of beneficial microbes like *Bifidobacterium* following prebiotic intake can lead to decreased alpha diversity, yet simultaneously enhance other health indicators such as immune function or metabolic health [43]. Collectively, these findings imply that changes in alpha diversity observed following ML supplementation in our study should be interpreted in conjunction with other health-related metrics rather than solely relying on microbial diversity indices.

SCFAs are crucial fermentation products generated by the gut microbiota. These metabolites play an essential role in maintaining intestinal homeostasis and supporting host physiological functions, including energy metabolism, immune regulation, and epithelial barrier integrity [10, 32]. Notably, SCFA levels can exhibit more pronounced differences than microbial composition due to their cumulative nature and reflection of functional metabolic outputs, as previously noted in comparative microbiome-metabolome studies [44, 45]. In this study, treatment with ML led to enhanced SCFA production during *in vitro* fecal fermentation, suggesting its fermentability and potential to support metabolically active microbial responses comparable to those observed with FOS. Alongside this metabolic response, shifts in microbial composition were also observed, notably the enrichment of taxa such as *Bifidobacterium*, Bacteroides, and Prevotella, which are known contributors to SCFA biosynthesis [39, 46]. *Bifidobacterium* predominantly produces acetate through saccharolytic metabolism and contributes to propionate production through the succinate pathway [46]. *Bifidobacterium* spp. utilize the fructose-6-phosphate phosphoketolase pathway to convert carbohydrates into acetate and lactate as primary end-products [47]. These fermentation products lower the pH of the gut environment and can act as cross-feeding substrates for butyrate-producing bacteria such as *Roseburia* and *Eubacterium* [48]. Bacteroides species express diverse polysaccharide utilization loci (PULs) that enable them to degrade complex carbohydrates such as dietary β-glucans and resistant starch, producing succinate as a metabolic intermediate. This is subsequently converted to propionate *via* the vitamin B_12_-dependent methylmalonyl-CoA pathway [49]. Among these, *B. uniformis*, which was enriched during fermentation with ML, expresses β-glucan-targeting PULs including glycoside hydrolases and SusC/D-like transport systems, facilitating the utilization of β-glucans and leading to SCFA production [50]. *P. copri*, another species that increased following fermentation with ML, contains MLG-PULs (mixed-linkage β-glucan polysaccharide utilization loci) that enable it to degrade cereal-derived β-glucans and generate succinate and propionate [51]. In dietary contexts rich in fiber, *P. copri* abundance has been positively correlated with increased SCFA production and improved gut metabolic health. Consistent with this hypothesis, the presence of *Roseburia* after fermentation suggests that butyrate production may have occurred through cross-feeding with acetate-producing bacteria. Acetate generated by *Bifidobacterium* can serve as a substrate for butyrate synthesis *via* the butyryl-CoA:acetate CoA-transferase pathway [52]. Fermentation of structurally complex substrates such as sake lees, rice bran, and β-glucans has similarly been shown to enhance SCFA production in both *in vitro* and *in vivo* models [36, 38, 46]. For instance, soluble and insoluble rice bran fiber fractions have been reported to differentially promote acetate and butyrate synthesis, while human intervention trials demonstrated that rice bran intake increases propionate and total SCFA levels [46, 53]. Barley-derived β-glucans have also been shown to improve host metabolism by enhancing microbial SCFA production in high-fat diet mouse models [44]. ML contain a substantial amount of indigestible substrates that can reach the colon and serve as fermentation substrates for gut microbiota. These residuals likely include rice-derived β-limit dextrins and resistant starch, yeast-derived β-glucans, and resistant proteins. Notably, these compounds resemble key components of established prebiotics such as FOS in their fermentability and capacity to selectively stimulate beneficial microbes. These compounds are known to function as fermentable substrates for *Bacteroides*, *Ruminococcus*, *Eubacterium*, and *Clostridium*, which degrade them into fermentation products such as acetate, propionate, and butyrate [54-56]. In particular, glutamate derived from protein fermentation can undergo acetogenesis and further support the growth of butyrate-producing bacteria such as *Faecalibacterium prausnitzii*. Collectively, these findings suggest that ML can serve as a metabolically active and structurally complex substrate that promotes SCFA biosynthesis through coordinated microbial activity involving saccharolytic, proteolytic, and cross-feeding pathways.

The results of *in vitro* digestion further support the fermentability and prebiotic potential of ML. A substantial portion of carbohydrates and proteins *Bifidobacterium*, *Bacteroides*, and *Prevotella* resistant components such as β-limit dextrins, resistant starch, and fermentation-resistant proteins likely reached the colon intact and served as microbial fermentation substrates [57, 58]. Makgeolli and sake are both produced using Japonica rice and undergo fermentation involving *Aspergillus oryzae* and *Saccharomyces cerevisiae* [3, 59]. Given this similarity in raw materials and microbial fermentation, compositional resemblance between their lees is plausible. Although analytical data specific to ML are limited, sake lees have been reported to contain approximately 14.3 g of resistant protein and 5.1 g of β-glucan per 100 g [60]. These β-glucans are derived from yeast cell walls, which consist of β-1,3-glucan backbones with β-1,6-linked side chains. The reduced protein digestibility observed in sake lees (<50%) has been attributed to the incorporation of protein into the yeast cell wall matrix, which interferes with enzymatic access [60, 61]. In consideration of these findings, ML are presumed to contain rice-derived indigestible polysaccharides, including β-limit dextrins and resistant starch, as well as microbial structural components. During traditional production, makgeolli is not subjected to fine-mesh filtration, resulting in the retention of whole yeast cells. The yeast cell wall is composed primarily of β-glucans and mannoproteins, which are resistant to host enzymatic digestion and can serve as fermentation substrates for intestinal microbes [62, 63]. These compositional features account for the resistance to digestion observed *in vitro* and highlight the potential of ML to support microbial fermentation and SCFA production in the colon.

The present study demonstrated the prebiotic potential of ML using an *in vitro* digestion and fecal fermentation system. However, several limitations should be considered when interpreting the results. First, the *in vitro* fermentation system does not fully replicate physiological conditions of the human gut, such as host-microbe interactions, immune responses, and intestinal motility. Although fecal material was standardized by pooling samples from multiple donors, individual microbial signatures may have still contributed to variation in fermentation outcomes. Further validation using *in vivo* models and diverse donor groups is necessary to confirm the health relevance of the observed effects. Second, although this study examined microbial composition and SCFA production, further investigations are needed to elucidate the underlying molecular mechanisms. In particular, metagenomic and metabolomic analyses could identify carbohydrate-active enzymes responsible for substrate degradation, clarify the metabolic pathways involved in SCFA biosynthesis, and uncover microbial cross-feeding interactions that contribute to fermentation dynamics. Third, the composition of ML may vary depending on fermentation parameters. This variation could affect reproducibility and functional efficacy. While a commercially manufactured product was used to maintain consistency, differences between brands or production conditions could still influence the outcomes. Future studies should examine how raw materials, fermentation duration, and microbial starters affect the nutritional profile and prebiotic activity of ML. Standardizing components such as β-glucan and resistant starch may help improve reproducibility across batches.

Beyond these limitations, the application of ML as a sustainable prebiotic ingredient presents an opportunity for industrial valorization. ML can be reprocessed into dietary supplements, fiber-enriched foods, and functional beverages. As demonstrated in the case of sake lees used in meat alternatives [7], ML may serve as a source of non-digestible carbohydrates and functional proteins. To realize their full potential in food and health industries, future research should also address safety evaluation, regulatory approval, and consumer acceptance. Taken together, the present findings provide scientific evidence supporting the development of ML as an eco-friendly, functional food material that contributes to both human health and resource sustainability.

## Conclusion

This study demonstrated that makgeolli lees (ML) exert prebiotic effects by modulating gut microbial composition, enhancing microbial diversity, and increasing the production of health-associated metabolites such as SCFAs. These effects are attributed to the structural complexity and fermentability of indigestible components including fungal β-glucans, resistant starch, and microbial residues retained in the lees. The findings highlight the potential of ML as a functionally relevant, upcycled dietary substrate capable of improving gut health. Given their abundance and limited current use, ML offer promising opportunities for incorporation into prebiotic formulations, functional foods, and sustainable fermentation-based dietary applications.

## Supplemental Materials

Supplementary data for this paper are available on-line only at http://jmb.or.kr.



## Figures and Tables

**Table 1:** Component of makgeolli lees before and after *in vitro* digestion.

Components	Before (g)	After (g)	Reduction rate (%)
Total carbohydrate	14.59 ± 1.74	6.85 ± 0.44	38.70 ± 6.52
Crude protein	4.82 ± 0.04	2.81 ± 0.03	10.08 ± 0.15
Crude fat	0.16	0.13	0.15
Total mass	20.00	12.17	

Total carbohydrate, crude protein, and crude fat contents were measured to assess compositional changes following *in vitro* digestion. Total carbohydrate content was determined by the phenol-sulfuric acid method, crude protein by the Kjeldahl method, and crude fat by Soxhlet extraction. Fat analysis was conducted in a single replicate. The total mass includes the weight of ash and represents the dry weight basis used for proximate analysis.

**Table 2:** Quantitative delta values of species level microbial changes classified as health-associated (H^+^) or health-negative (H^-^) following fructooligosaccharide or makgeolli lees.

Health effect	Microorganism	Δ Relative abundance (%)	
		Fructooligosaccharide	Makgeolli lees
H (+)	*Prevotella copri*	1.03 ± 0.28	0.98 ± 0.24
	*Megamonas funiformis*	0.70 ± 0.27	-0.91 ± 0.04
	*Bifidobacterium catenulatum*	0.63 ± 0.73	-0.77 ± 0.30
	*Bifidobacterium bifidum*	0.57 ± 0.41	0.37 ± 0.09
	*Ruminococcus torques*	0.15 ± 0.02	0.03 ± 0.03
H (-)	*Bacteroides vulgatus*	1.29 ± 0.64	2.49 ± 0.46
	*Clostridium leptum*	0.13 ± 0.03	0.05 ± 0.05
	*Bacteroides plebeius*	-0.03 ± 0.11	0.05 ± 0.05
	*Lachnoclostridium* spp.	-0.06 ± 0.04	-0.09 ± 0.12
	*Bacteroides coprocola*	-0.55 ± 1.25	4.55 ± 0.26

Health-promoting microorganisms are represented as H(+), while harmful microorganisms are represented as H(-), following the methodology described in Chang *et al*. (2024), the notified species in the Ministry of Food and Drug Safety, and various references. Error bars indicate the standard deviation of triplicate experiments. In addition, it shows how the relative abundance of microorganisms in fructooligosaccharide (FOS) and makgeolli lees (ML) lees samples changed after 12 h of fermentation.

**Table 3:** Changes in metabolites concentration (mM) during *in vitro* fecal fermentation after 12 and 24 h.