# Supplementary material for: Makgeolli Lees as a Novel Prebiotic Candidate: Effects on Human Gut Microbiota and Metabolites
Source: J Microbiol Biotechnol. 2025 Jun 23;35:e2504020. doi: 10.4014/jmb.2504.04020 (PMC12256836; doi:10.4014/jmb.2504.04020)
Supplement: Supplementary file 1 [file jmb-35-e2504020-supple.pdf]

J. Microbiol. Biotechnol. 2025. 35: e2504020

https://doi.org/10.4014/jmb.2504.04020

Supplementary Table S1. Quantitative delta values of species level microbial changes classified as health-associated (H^+) or health-negative (H^-) following FOS or ML.

Health effect	Microorganism	Δ Relative abundance (%)	
		Fructooligosaccharide	Makgeolli lees
H (+)	*Prevotella copri*	1.03 ± 0.28	0.98 ± 0.24
	Megamonas funiformis	0.70 ± 0.27	-0.91 ± 0.04
	Bifidobacterium catenulatum	0.63 ± 0.73	-0.77 ± 0.30
	Bifidobacterium bifidum	0.57 ± 0.41	0.37 ± 0.09
	Ruminococcus torques	0.15 ± 0.02	0.03 ± 0.03
	Alistipes onderdonkii	0.07 ± 0.07	0.05 ± 0.05
	Holdemania	0.05 ± 0.05	0.01 ± 0.02
	Lactobacillus gasseri	-0.02 ± 0.04	-0.56 ± 0.22
	Alistipes shahii	-0.05 ± 0.05	0.06 ± 0.05
	Bacteroides uniformis	-0.08 ± 0.07	0.46 ± 0.06
	Flavonifractor	-0.09 ± 0.06	-0.10 ± 0.04
	Odoribacter splanchnicus	-0.10 ± 0.01	0.07 ± 0.11
	Ruminococcus	-0.14 ± 0.07	-0.01 ± 0.09
	Bacteroides cellulosilyticus	-0.14 ± 0.02	0.06 ± 0.07
	Ruminococcus bicirculans	-0.17 ± 0.12	0.16 ± 0.06
	Megasphaera	-0.20 ± 0.00	-0.24 ± 0.12
	Coprococcus comes	-0.22 ± 0.24	-0.02 ± 0.29
	Akkermansia muciniphila	-2.16 ± 0.28	-1.80 ± 0.45
	Dorea formicigenerans	-2.35 ± 0.54	-1.96 ± 0.21
	Intestinibacter	-6.94 ± 0.62	-6.92 ± 0.53
H (-)	*Bacteroides vulgatus*	1.29 ± 0.64	2.49 ± 0.46
	Clostridium leptum	0.13 ± 0.03	0.05 ± 0.05
	Bacteroides plebeius	-0.03 ± 0.11	0.05 ± 0.05
	Lachnoclostridium	-0.06 ± 0.04	-0.09 ± 0.12
	Bacteroides coprocola	-0.55 ± 1.25	4.55 ± 0.26
	Bacteroides ovatus	-4.11 ± 1.34	-2.14 ± 1.51

Health-promoting microorganisms are represented as H(+), while harmful microorganisms are represented as H(-), following the methodology described in Chang et al. (2024), the notified species in the Ministry of Food and Drug Safety, and various references. Error bars indicate the standard deviation of triplicate experiments. In addition, it shows how the relative abundance of microorganisms in fructooligosaccharide and Makgeolli lees samples changed after 12 h of fermentation.
